# Copy Number Variants Associated with 14 Cases of Self-Injurious Behavior

**DOI:** 10.1371/journal.pone.0149646

**Published:** 2016-03-02

**Authors:** Matthew D. Shirley, Laurence Frelin, José Soria López, Anne Jedlicka, Amanda Dziedzic, Michelle A. Frank-Crawford, Wayne Silverman, Louis Hagopian, Jonathan Pevsner

**Affiliations:** 1 Program in Biochemistry, Cellular and Molecular Biology, Johns Hopkins School of Medicine, Baltimore, Maryland, United States of America; 2 Department of Neurology, Hugo W. Moser Research Institute at Kennedy Krieger, Baltimore, Maryland, United States of America; 3 Johns Hopkins School of Medicine, Baltimore, Maryland, United States of America; 4 Genomic Analysis and Sequencing Core, Johns Hopkins Bloomberg School of Public Health, Baltimore, Maryland, United States of America; 5 Deptartment of Behavioral Psychology, Kennedy Krieger Institute, Baltimore, Maryland, United States of America; 6 Department of Psychiatry and Behavioral Sciences, Johns Hopkins School of Medicine, Baltimore, Maryland, United States of America; IGBMC/ICS, FRANCE

## Abstract

Copy number variants (CNVs) were detected and analyzed in 14 probands with autism and intellectual disability with self-injurious behavior (SIB) resulting in tissue damage. For each proband we obtained a clinical history and detailed behavioral descriptions. Genetic anomalies were observed in all probands, and likely clinical significance could be established in four cases. This included two cases having novel, *de novo* copy number variants and two cases having variants likely to have functional significance. These cases included segmental trisomy 14, segmental monosomy 21, and variants predicted to disrupt the function of *ZEB2* (encoding a transcription factor) and *HTR2C* (encoding a serotonin receptor). Our results identify variants in regions previously implicated in intellectual disability and suggest candidate genes that could contribute to the etiology of SIB.

## Introduction

Self-injurious behavior (SIB) is a serious behavioral disorder exhibited by many individuals with autism spectrum disorder (ASD) and other forms of developmental disability. It is characterized by self-inflicted physical injury to one's own body [1], with common topographies of SIB including head banging on hard surfaces, head and body hitting, self-biting, eye poking, self-scratching, and hair pulling. The prevalence of SIB is estimated at 5 to 17% of persons with intellectual disability [2], and approximately 50% of individuals displaying SIB are also diagnosed with ASD [3]. Left untreated, SIB usually persists and evolves to include further topographies of injury and often has significant adverse effects on child and family quality of life.

SIB likely has a multifactorial etiology, with some portion of SIB cases arising from an interaction between genetic and environmental factors during development. Prior research has established that single gene mutations cause syndromes with increased risk for SIB as part of the phenotype, including Lesch-Nyhan syndrome [4], Rett syndrome [5], Fragile X syndrome [6], and the Cornelia de Lange syndrome [7]. In some cases copy number variants (CNVs) affecting these single genes also have causal roles [6]. We hypothesized that structural variants such as chromosomal deletions and duplications may also underlie some nonsyndromic cases of SIB in individuals with ASD.

In this study we recruited participants (probands and family) from the Neurobehavioral Unit (NBU) at the Kennedy Krieger Institute, an inpatient unit for children with disabilities and severe behavior disorders. We applied high-density single nucleotide polymorphism (SNP) microarray analysis to DNA derived from these patients. The present study was restricted to children with ASD together with SIB. Based on previous findings of genetic factors associated with both ASD and SIB we hypothesized that this population represents an enrichment of single nucleotide variants (SNVs), deletions, and duplications contributing to the SIB phenotypes. Given the heterogeneity of SIB phenotypes, we expected that the variants we identified could each account for only one specific case of SIB. Due to the expected limitations in associating recurrent variants with phenotypes, we applied widely adopted criteria to aid in identifying potentially causal alterations [8].

## Materials and Methods

All studies were performed with written informed consent and approval of the Institutional Review Board at Johns Hopkins University. Probands were recruited from the NBU at the Kennedy Krieger Institute.

### Inclusion Criteria

Probands with an ASD and at least one topography of SIB were recruited for enrollment. Autism diagnosis was confirmed by a child psychiatrist upon admission to the NBU. Probands had at least one biological parent available for additional genetic analyses.

### Vineland Adaptive Behavior Scales

Measures of adaptive behavior were obtained from parents for 11 of the 14 probands using the Vineland Adaptive Behavior Scales (Vineland)[9]. While other instruments generally relevant to autism are available, such as the revised Autism Diagnostic Interview (ADI-R) or Autism Diagnostic Observation Schedule (ADOS), those measures are not routinely used for the population with severe behavioral disturbances. The first or second edition of the Vineland [9] was administered depending on the time of admission to the inpatient hospital. Both editions of the Vineland measure adaptive behavior across three domains: Communication (i.e., expressive, receptive, and written language skills), Daily Living Skills (i.e., personal, domestic, and community-based skills), and Socialization (i.e., interpersonal relationships, play and leisure, and coping skills). The second edition also includes a Motor Skills domain (i.e., fine and gross motor skills). Raw scores from each domain are converted into standard scores (M = 100, SD = 15) and an adaptive behavior composite score (M = 100, SD = 15). In addition, the standard scores within each domain and the adaptive behavior composite are categorized as one of five levels of adaptive functioning: low, moderately low, adequate, moderately high, and high.

### Functional behavioral assessment

Functional analyses [10] of SIB were conducted for all participants to identify the variables that evoke and maintain SIB. The functional analysis is part of the standard of care for all patients admitted to the NBU; and for assessment of SIB, in general. During the standard functional analysis, between four and six conditions (i.e., alone, attention, demand, tangible with toys, and/or tangible with food, and toy play), each typically lasting 10 minutes, are presented in a multi-element (or alternating treatments) design. Each test condition is designed to simulate an environment the individual is likely to encounter, with variables hypothesized to give rise to and maintain problem behavior manipulated systematically. Responding in those conditions is compared to a control condition simulating an enriched environment where attention and toys are freely available. Behavioral data were graphically depicted and interpreted using criteria such as those developed by Hagopian et al. that consider magnitude of effect, stability, and trend [11].

### Sample collection and DNA extraction

We obtained blood samples from each proband, and from each parent or sibling we obtained a blood or saliva sample. We isolated DNA from each participant using a PureGene kit (QIAGEN, Valencia, CA). Lymphoblast cell lines were established from some blood samples for the purpose of performing fluorescence in-situ hybridization.

### SNP arrays

DNA samples were assayed using the Affymetrix GenomeWide 6.0 platform at the Johns Hopkins Genomic Analysis and Sequencing Core (Baltimore, MD). Genotyping was performed using the birdseed-v2 algorithm [12]. Data have been deposited in the Database of Genotypes and Phenotypes (dbGaP) at the National Institutes of Health (http://www.ncbi.nlm.nih.gov/projects/gap/cgi-bin/study.cgi?study_id=phs000337.v1.p1).

### CNV Analysis

Copy number segmentation was performed using PennCNV [13]. Only samples with a logR ratio standard deviation of less than 0.4 were analyzed. In families where trios (proband and both biological parents) were available, the PennCNV joint trio Hidden Markov Model (HMM) was used to incorporate copy number from the entire trio. Using an R script, copy number variants were annotated by intersection with Database of Genomic Variants [14], NCBI RefSeq genes [15], and The Centre for Applied Genomics Autism Chromosome Rearrangement Database (TCAG-ACRD)[16]. We defined a CNV as a hemizygous or homozygous deletion, amplification of three or more copies, and greater than 20 kilobases.

### Criteria for inclusion of candidate loci

Based on guidelines developed by the American College of Medical Genetics (ACMG) [8], uniform criteria to define CNVs of potential significance were applied. That group provided evidence-based analysis showing that for individuals with unexplained ID, ASD, or multiple congenital abnormalities (MCAs), microarrays offer greater diagnostic yield (15–20%) for genetic testing than karyotyping. Our seven primary criteria indicating that a CNV is possibly pathogenic are that: (1) a CNV is altered relative to unaffected parents; (2) a CNV is similar to one in an affected relative; (3) the CNV is not completely contained within a region of genomic imbalance as defined by a CNV database of healthy individuals, employing a similarly high-resolution technology; (4) a CNV overlaps a genomic imbalance in a database of probands with ID/DD, ASD, or MCAs; (5) a CNV overlaps a locus for a known genomic imbalance syndrome; (6) a CNV contains morbid Online Inheritance in Man (OMIM) genes; or (7) a CNV is gene rich. The International Standards for Cytogenomic Arrays (ISCA) criteria were also consistent with those of the ACMG [17]. Both the ISCA and ACMG sets of guidelines represent strategies that help interpret whether variants are pathogenic, benign, or of unknown significance. As many of these sources of evidence as possible were applied to each interpretation in this study. For example, for cases in which only one parent was available it was not always possible to confirm whether particular CNVs were inherited or de novo.

### FISH

Fluorescence in-situ hybridization (FISH) was performed using fosmids obtained from the BACPAC Resource Center (Children's Hospital Oakland Research Institute). These were G248P8731F5 (chr2:145,173,324–145,216,003 spanning 42,680 bp in GRCh37) and G248P85468F6 (chr2:145,516,578–145,557,736 spanning 41,159 bp in GRCh37). Fosmids were nick translation labeled with spectrum green or orange dUTP (Abbott Molecular Diagnostics) and hybridized to lymphoblastoid cell lines. Local copy number was determined by counting fluorescence signals from metaphase and interphase cells.

### Database analyses

We accessed the DECIPHER database (v5.1, GRCh37 accessed on March 8, 2012). The DECIPHER project includes information on patients with chromosomal microdeletions and microduplications [18]. We obtained a comprehensive list of cases and chromosomal loci involving those areas relevant to the current analysis. Of these, entries were excluded having a classification of “Familial inherited from normal parent” or “Unknown—parents not analyzed/uninformative.”

## Results

Demographic and clinical summaries of the 14 studied probands are given in Table 1. The ages of probands, of whom 12 (~86%) were male, ranged from 6 to 17 years, with an average age of 12. For the sample of children included in the current study for whom the Vineland was conducted, scores in the Communication, Daily Living Skills, and Socialization domains all fell within the low range of adaptive functioning. Similarly, all children scored in the low range of adaptive functioning for the Adaptive Behavior Composite. For the Motor Skills domain (assessed with Probands 7, 8, 10, 11, 12, 13, and 14), 57.1% of the sample fell within the low range, 28.6% fell within the moderately low range, and 14.3% fell within the adequate range. Table 2 depicts the standard scores across domains.

**Table 1:** Demographic and clinical summary of probands.

Proband	Age	Sex	Psychiatric and Medical Diagnoses
**1**	13	F	Autism spectrum disorder (ASD), disruptive behavior disorder not otherwise specified (DBD NOS), stereotypic movement disorder with self-injurious behavior (SMD with SIB), moderate intellectual disability (ID), seizure disorder.
**2**	16	M	ASD, severe ID, DBD, SMD with SIB, and a history of Stevens Johnson Syndrome
**3**	12	M	ASD, severe ID, SMD with SIB, unspecified disturbance of conduct, and impulsive control disorder NOS
**4**	9	M	ASD, Severe ID, agenesis of the corpus callosum, cerebral palsy, and thrombocytopenia
**5**	10	M	ASD and moderate ID
**6**	17	M	ASD, SMD with SIB, unspecified disturbance of conduct, and severe ID
**7**	13	M	ASD, SMD with SIB, DBD NOS, obsessive compulsive disorder, bipolar affective disorder, and unspecified level of ID
**8**	14	M	ASD, SMD with SIB, DBD NOS, and history of multiple small bowel ulcers of unclear etiology
**9**	12	M	ASD, SMD with SIB, and unspecified disturbance of conduct
**10**	13	F	ASD, SMD with SIB, DBD NOS, Mood Disorder NOS, and profound ID
**11**	6	M	ASD, DBD NOS, SMD with SIB and moderate ID
**12**	9	M	ASD, DBD, SMD with SIB, mood disorder, and impulse control disorder
**13**	17	M	ASD, DBD (NOS), severe ID, SMD with SIB, and mood disorder (NOS)
**14**	11	M	ASD, obsessive-compulsive disorder, moderate ID, and unspecified mood disorders

**Table 2:** Standard scores from Vineland and Vineland-II for each domain and the adaptive behavior composite.

Proband	Communication	Daily Living Skills	Socialization	Motor Skills	Adaptive Behavior Composite
**1**	<20	<20	<20	N/A	<20
**4**	54	48	53	N/A	52
**6**	<20	<20	<20	N/A	<20
**7**	45	50	45	94	46
**8**	48	36	46	59	38
**9**	57	57	50	N/A	55
**10**	34	35	38	56	33
**11**	42	40	53	59	46
**12**	38	40	48	30	39
**13**	33	43	48	78	40
**14**	53	59	40	81	51

Following interviews with caregivers and direct observation of the targeted problem behaviors, behavioral staff members established distinct operational definitions for each topography for each patient. All participants were reported to engage in at least two topographies of SIB (mean = 3.9, range = 2–7 topographies). Similar to the results obtained by Kahng et al. [19], the most commonly observed topographies of SIB included hitting one’s head with an open or closed fist or object, biting oneself (often targeting the hands, wrists, or arms), and banging one’s head against surfaces. Head-hitting, self-biting, and head-banging were reported to occur in 86%, 71%, and 64% of all 14 cases, respectively (S1 Table). We identified the targeted body regions when participants engaged in SIB, other problem behavior, and the functional analysis outcomes for all topographies of SIB (S2 Table).

We determined the percentage of functional analysis outcomes for each reported topography of SIB (n = 55) (S2 Table). It should be noted that some topographies may have been counted in more than one functional analysis outcome category if the topography was observed to have multiple functions (e.g., if head-banging were observed to be maintained by access to attention and to preferred toys, it would be counted in the Attention, Tangible–Toy, and Multiply Maintained categories). Functional analysis results for SIB are summarized in Table 3.

**Table 3:** Participant SIB topographies and functional analysis outcomes.

Proband	SIB topography	Functional analysis outcome
**Proband 1**	Head-hitting, self-biting, body-hitting, chin-banging	Multiply maintained: Escape from demands and access to tangible stimuli
**Proband 2**	Head-hitting, self-biting, head-banging, body-hitting, self-pinching, self-scratching, hand/wrist-banging	Multiply maintained: Access to edible stimuli and access to attention
**Proband 3**	Head-hitting, self-biting, hand/wrist-banging	Automatic reinforcement
	Head-banging	Access to edible stimuli
**Proband 4**	Head-hitting, self-biting, head-banging	Automatic reinforcement
	Head-banging on body (including foot, knee, leg)	Low to zero rates observed in FA, function not identified
**Proband 5**	Head-hitting	Multiply maintained
	Self-biting	Low to zero rates observed in FA, function not identified
**Proband 6**	Head-banging	Automatic reinforcement
	Head-hitting, self-biting	Escape from demands
**Proband 7**	Head-hitting, self-biting, body-hitting, self-scratching	Low to zero rates observed in FA, function not identified
**Proband 8**	Self-biting, head-banging	Multiply maintained: Access to tangible stimuli and automatic reinforcement
	Head-hitting, self-scratching, self-pinching	Automatic reinforcement
**Proband 9**	Head-hitting, body-hitting, hair-pulling, chin-banging/pressing	Multiply maintained: Access to attention, escape from demands, automatic reinforcement
	Head-banging	Low to zero rates observed in FA, function not identified
**Proband 10**	Head-hitting, head-banging, body-hitting, self-pinching, eye-SIB	Automatic reinforcement
**Proband 11**	Head-hitting, head-banging	Low to zero rates observed in FA, function not identified
**Proband 12**	Self-biting, hair-pulling	Low to zero rates observed in FA, possible access to attention
**Proband 13**	Head-hitting, body-hitting, skin-picking, self-scratching, body-slamming	Low to zero rates observed in FA, function not identified
**Proband 14**	Self-biting, body-hitting	Access to attention
	Head-banging	Automatic reinforcement

Abbreviation: FA, functional analysis.

Genotyping was completed for a total of 40 individuals, including: (1) three father, mother, proband, and sibling quartets (Probands 1, 4, 9), (2) five father, mother, and proband trios (Probands 3, 7, 10, 12, 14), (3) one father, proband, and sibling trio (proband 6), and (4) five parent/child pairs (Probands 2, 5, 8, 11, 13). To confirm that the pedigree structures were correct as obtained from family interviews, and to identify large-scale chromosomal changes, we analyzed the genotype data using identity-by-state (IBS) and identity-by-descent (IBD) methods. This allowed us to estimate Cotterman coefficients of relatedness of all individuals in the study. We analyzed all pairwise relationships between the 40 individuals in this study (n = 780 pairwise comparisons) using kcoeff software to analyze autosomal SNP data [20] (data not shown). This confirmed the expected number of parent-child relationships (n = 29), full siblings (n = 4), and unrelated individuals and allowed us to confirm that all 14 pedigrees were correct as annotated. We noted a slight decrease in IBD1 (with a corresponding increase in IBD0) between proband 4 and mother, consistent with a chromosomal deletion (see below).

For four of the 14 cases (Probands 1–4) we identified a CNV likely to have a causal role. We describe these in the following paragraphs. For Probands 5–14 we identified CNVs that are less likely to have causal roles because they are: (a) inherited from a normal parent, (b) located in genomic regions lacking disease-associated genes, or (c) at locations lacking any genes. In other cases only one parent was available for this study, making conclusive interpretation of genetic findings difficult and these cases were classified as negative to be consistent with conservative criteria for identifying likely causal alterations. However, the possibility of some of these alterations being causal cannot be ruled out definitively, and the true percentage of positive cases could be higher. For Probands 5–14 we describe clinical and behavioral phenotypes as well as CNVs in S1 File.

**Proband 1.** The proband was a female reported to have global developmental delay at 6 months of age with failure to meet milestones and spastic quadraparesis and was subsequently diagnosed with autism and developmental delay. Proband 1’s SIB included head-hitting, self-biting, body-hitting, and chin-banging. Results of her functional analysis suggested that her SIB was multiply maintained by escape from demands and access to preferred toys.

**Fig 1:**
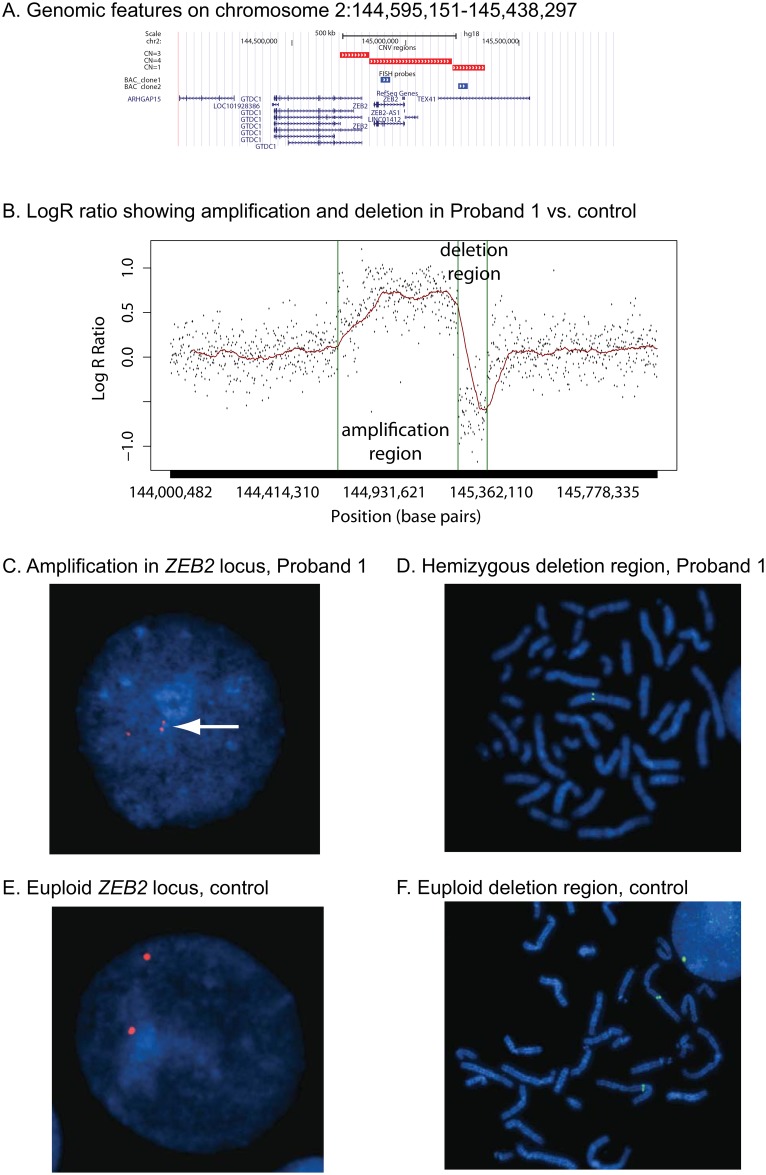
Evidence of a tandem amplification and heterozygous deletion at the *ZEB2* locus in proband 1. (A) Genomic landscape (from the UCSC Genome Browser, GRCh36/hg18, a ~2 Mb region spanning chr2:144000482–145945938 corresponding to all SNPs on the Affymetrix array from 144–146 Mb). (B) LogR ratio plot of amplification/deletion region for proband 1 and control (from the GRCh36/hg18, chr2:144000482–145945938). Vertical bars indicate the beginning and end of the amplification and deletion regions. Red line indicates a moving average. (C) Representative example of interphase FISH with labeled fosmid G248P8731F5 using lymphoblast cell lines derived from proband 1 and (E) an unaffected control. Note the presence of three copies in the patient (panel C, white arrow indicating a duplication homolog). (D) Representative example of metaphase FISH using a probe from the deletion region (fosmid G248P85468F6). (F) Metaphase FISH in a euploid control reveals the expected two chromosomal copies of this fosmid probe.

CNV analysis indicated two contiguous copy number variations. The first was a de novo amplification spanning 491 kb on chromosome 2q22.3, immediately followed by a heterozygous deletion region (spanning 141 kb) (Fig 1A). These copy number changes were observed with a log R ratio plot (Fig 1B). We confirmed both the amplification and deletion by FISH. Amplification (3 signals) was observed in 64% of the interphase cells counted for the proband vs. 16% observed in the control (examples shown in Fig 1C and 1E). For the deletion we counted 10 metaphase cells, each of which had only one signal in the proband (example shown in Fig 1D) and two signals were observed in the control in 5 metaphase cells (example in Fig 1F). Affymetrix analysis software determined the amplified region was from two to three copies for 127 kb immediately followed by amplification to four copies for the remaining 364 kb (see Fig 1A). However the log R ratio plot (Fig 1B) and the FISH probe in the putative region of four copies both indicate that the amplification produced a total of three copies.

The amplification region overlapped with the *ZEB2* gene encoding zinc finger E-box binding homeobox 2, a DNA-binding transcriptional repressor. Loss-of-function mutations in *ZEB2* cause Mowat-Wilson syndrome, an autosomal dominant disorder characterized by intellectual disability, delayed motor development, and epilepsy. However, the proband’s dysmorphism was distinct from the characteristic features of Mowat-Wilson syndrome. Only deletions, inversions, and frameshift mutations in *ZEB2* have been reported in individuals with Mowat-Wilson syndrome. To this date, no amplifications of *ZEB2* have been reported in the literature.

**Proband 2.** The proband was a male diagnosed with ASD, severe intellectual disability, disruptive behavior disorder, and stereotypical movement disorder with self-injurious behavior. Proband 2 engaged in head-hitting, self-biting, head-banging, body-hitting, self-pinching, self-scratching, and hand/wrist banging. Results of his functional analysis suggested that his SIB was multiply maintained by access to preferred foods and access to attention. He had a history of Stevens-Johnson syndrome (OMIM #608579; susceptibility to severe cutaneous adverse reaction). A karyotype had been performed in 1994 at age 2, reported as 46,XY,-7,+der(7)t(7:14)(p22:q13)psu dic(14)(q13). This indicated a 7;14 translocation with trisomy for proximal 14q and possible monosomy for 7p.

**Fig 2:**
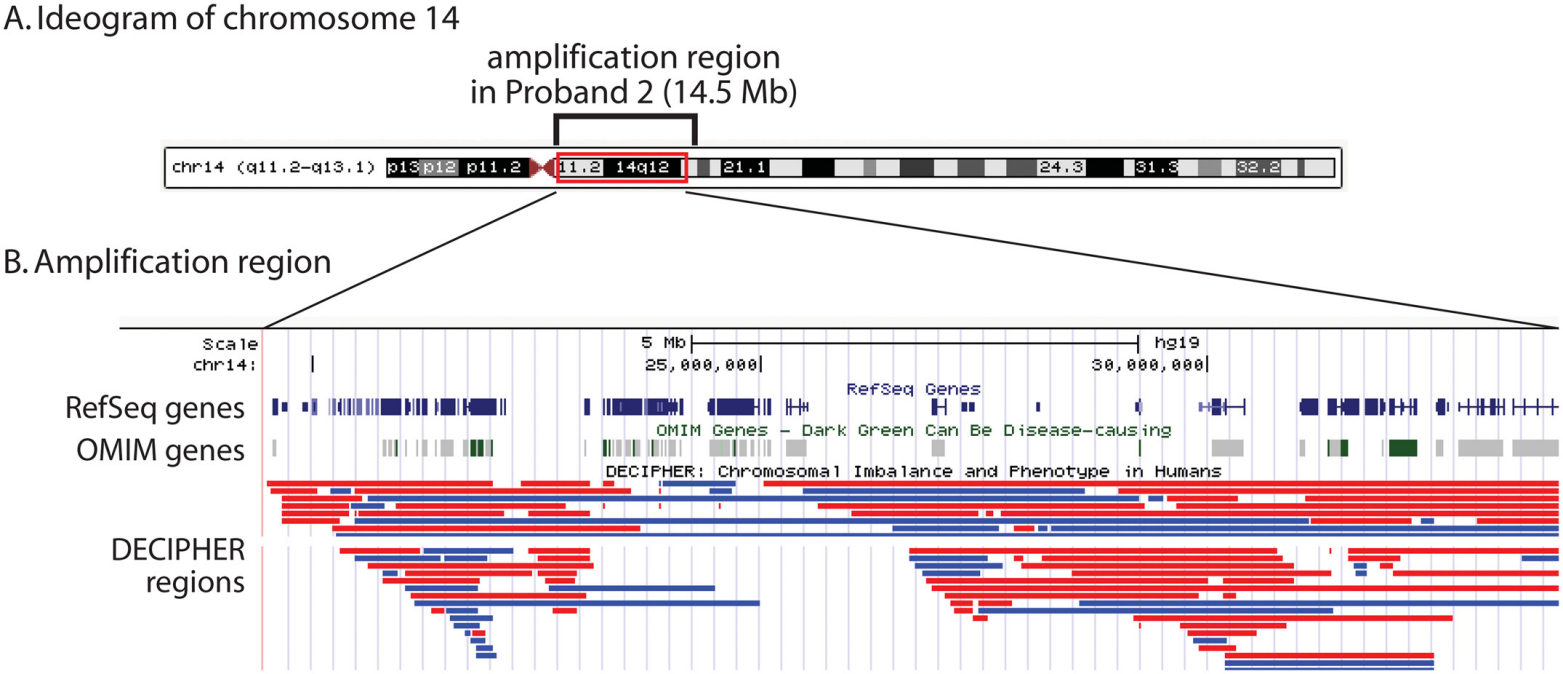
Region of segmental trisomy on chromosome 14 in proband 2. (A) The region spans 14.5 Mb (GRCh37/hg19 chr14:19,487,381–33,947,341). (B) The amplification region includes 404 RefSeq genes. Additional tracks show DECIPHER regions of chromosomal imbalances (deletions in red, duplications in blue) and OMIM disease genes.

Copy number segmentation, performed on the proband and his mother, indicated a large gain on 14q11.2-q13.1 from 19.4 to 34.0 Mb, spanning 14.6 Mb (Fig 2). This region included 256 RefSeq genes. Approximately 40 cases of mosaic trisomy 14 have been reported [21,22], but not the segmental trisomy observed in this proband. There were no corresponding duplications in the DECIPHER database. Trisomy 14 mosaicism is characterized by growth and psychomotor retardation, dysmorphic craniofacial features (e.g. broad nose, dysplastic ears, micrognathia), congenital heart anomalies, and genitourinary abnormalities [23]. Mental development is normal or near normal (e.g. [24]), and individuals with mosaic trisomy 14 have not been reported to display SIB-related behavior.

Other large CNVs included a 1.8 Mb amplification on chromosome 15q11.1-q11.2, a region associated with intellectual disability and developmental delay [25]; and a 131 kb hemizygous deletion on 22q11.21. This region spanned the DiGeorge syndrome critical region gene 5 and 6 (*DGCR5* and *DGCR6*) as well as the proline dehydrogenase 1 (*PRODH)* gene implicated in schizophrenia and hyperprolinemia type 1.

Stevens-Johnson syndrome has been associated with HLA-class I alleles on chromosome 6p22.1 and 6p21.33. We observed no CNVs at those loci, nor did we detect any copy number change consistent with chromosome 7 segmental monosomy.

**Proband 3.** The proband was a male exhibiting SIB resistant to medication treatment since age 2. Proband 3 engaged in head-hitting, self-biting, head-banging, and hand/wrist banging. Results of his functional analysis suggested that his head-hitting, self-biting, and hand/wrist banging were maintained by automatic reinforcement. Head-banging was found to be maintained by access to preferred foods. He demonstrated hundreds of self-injurious and aggressive behaviors hourly. Diagnoses included ASD, severe intellectual disability, stereotypic movement disorder with self-injury, unspecified disturbance of conduct, and impulse control disorder not otherwise specified. Over the course of six years he was placed on 28 medication trials but no medication afforded any significant sustained reduction in self-injury or aggression. Based on careful consideration of this history and restricted treatment options, the decision was made to consider electroconvulsive shock therapy. This was employed successfully, significantly reducing episodes of SIB with minimal side effects and contributed to healing and salvation of vision [26].

**Fig 3:**
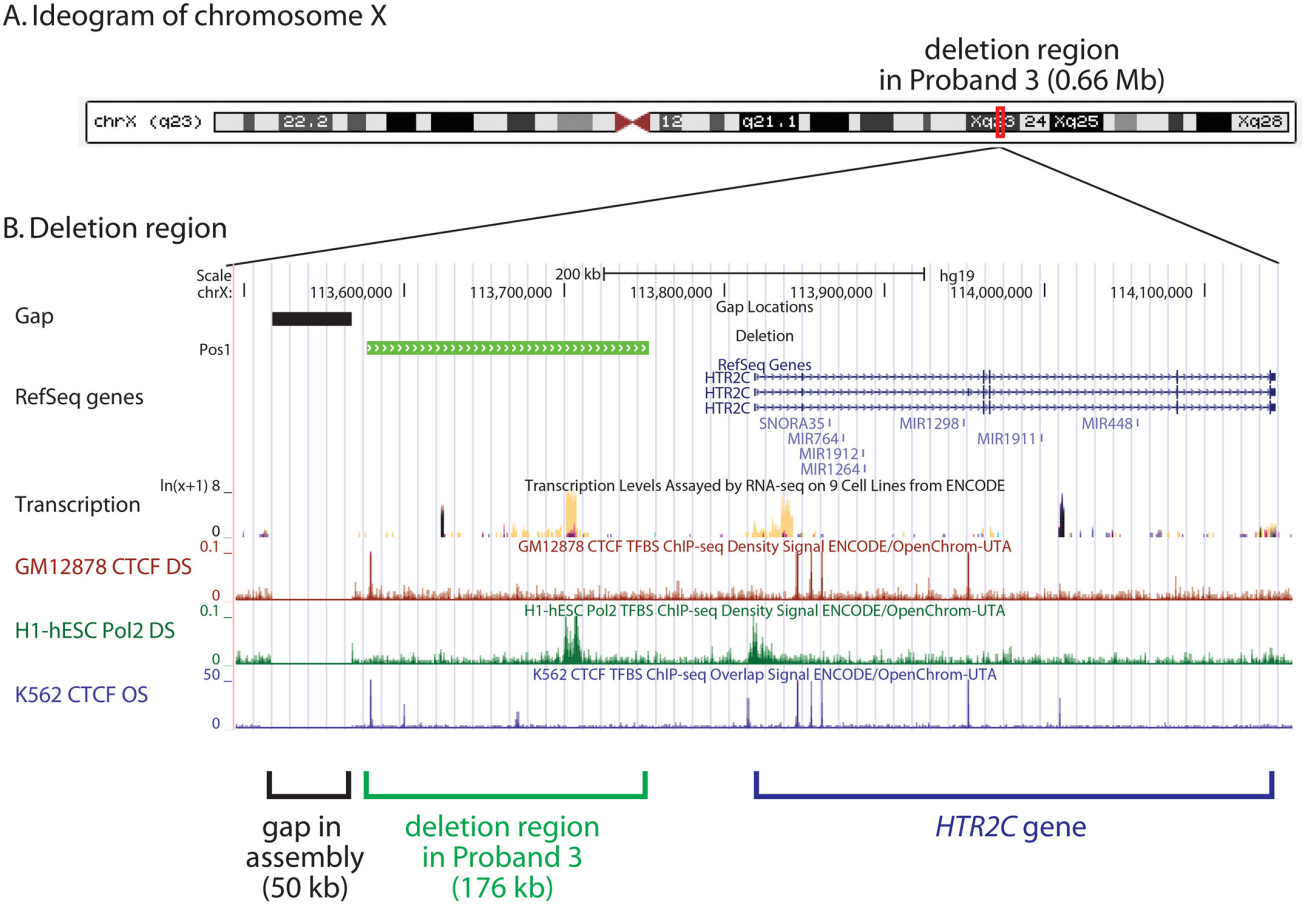
Hemizygous deletion in proband 3 spanning 176 kb in a regulatory region of the gene encoding an X-linked serotonin receptor, *HTR2C*. (A) An ideogram of chromosome X from the UCSC Genome Browser is shown, indicating the deletion site on Xq23. (B) The deletion region and surrounding landscape is shown, spanning 660,000 base pairs (chrX:113,495,001–114,155,000). The deletion region spans 106 SNPs (GRCh37/hg19 chrX:113,577,005–113,752,952). A 50 kb gap at the 3’ end of the deletion is indicated (chrX:113517669–113567668). Upstream regulatory elements are indicated displayed on tracks for transcription level from RNA-seq and transcription factor ChIP-seq.

We detected a *de novo* 176 kb hemizygous deletion on Xq23 upstream of the *HTR2C* gene encoding 5-hydroxytryptamine (serotonin) receptor 2C (Fig 3). Given the role of *HTR2C* in modulating synaptic transmission, this represents a candidate gene for the proband’s disease phenotype. The deletion region is flanked on the 5’ side by a gap in the GRCh38 assembly, spanning 70 kb. The deletion region resides in a potential regulatory region, extending 66 kb upstream of the 5’ end of the *HTR2C* gene. Within the deletion region, a CpG island corresponds to a DNase I hotspot and to a position strongly likely to have regulatory function based on data from the ENCODE project [27] (Fig 3).

**Proband 4.** The proband was an 8 1/2 year old male with severe intellectual disability, ASD, agenesis of the corpus callosum, cerebral palsy, and thrombocytopenia. Proband 4’s SIB included head-hitting, self-biting, head-banging (against hard surfaces), and head-banging against the foot, knee, or leg or other body parts. Head-hitting, self-biting, and head-banging against hard surfaces were observed to be maintained by automatic reinforcement.

**Fig 4:**
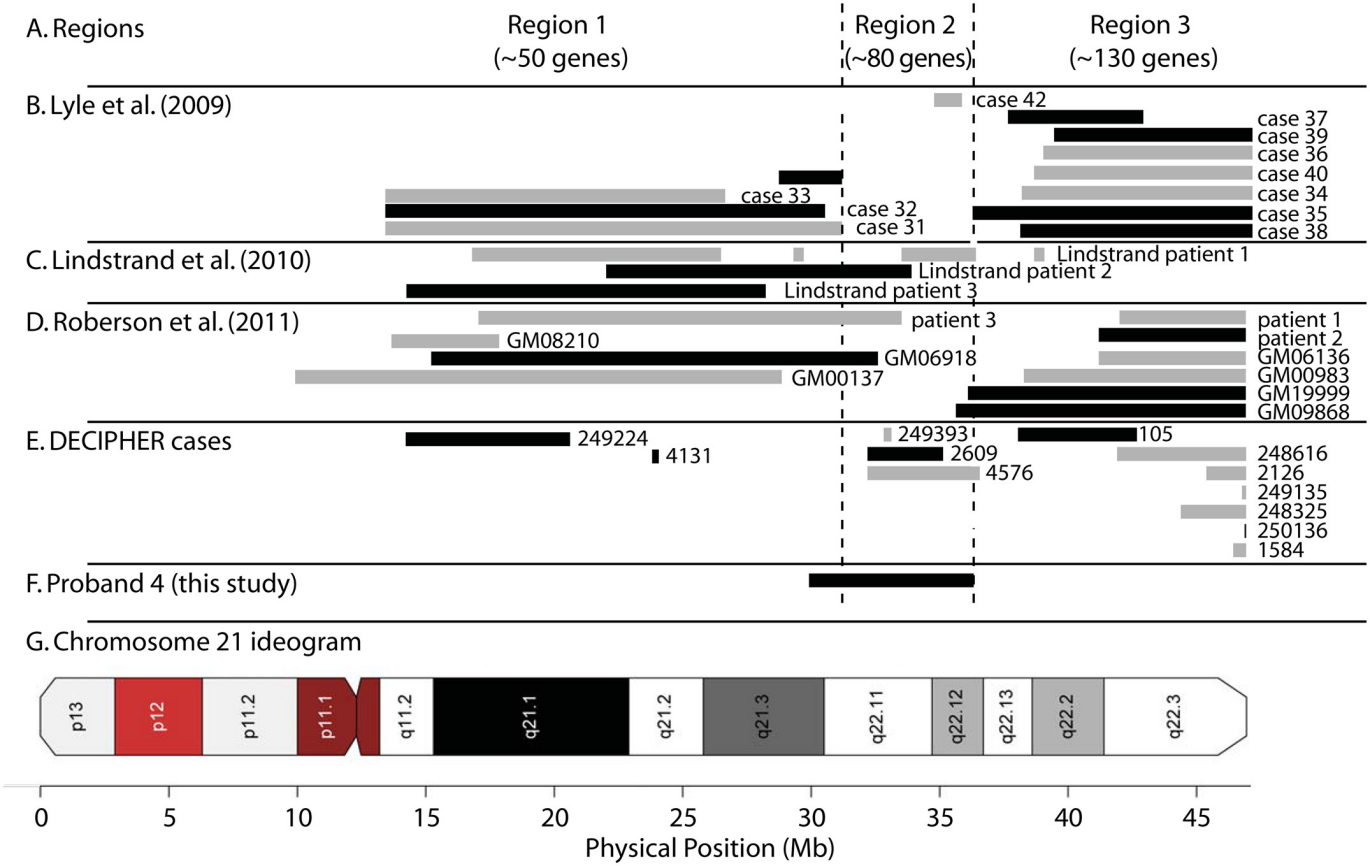
Heterozygous deletion of 6.5 Mb spanning a region of chromosome 21q in proband 4. This region is associated with agenesis of the corpus callosum. (A) We define three regions across the q arm of chromosome 21. (B) Extent of deletions reported by Lyle et al. [28]. (C) Three deletions reported by Lindstrand et al. [38]. (D) We previously reported chromosome 21 partial monosomy in three patients and additional cases from Coriell Repository [39]. (E) DECIPHER database cases spanning the chromosome. (F) Proband 4 (this study) has a hemizygous deletion that is notable for spanning the entirety of region 2, possibly accounting for the severe phenotype. (G) Chromosome 21 ideogram. This figure was adapted from [39] incorporating data on proband 4.

Chromosomal analysis revealed 21q segmental monosomy spanning 6.5 Mb (Fig 4F). Proband 4 and his mother had a Cotterman’s coefficient k1 value of 0.9950, while all other parent/child relationships had values of 1.0 ± 0.0002. This is consistent with the slight loss of IBD1 sharing that occurs when an individual has a heterozygous chromosomal deletion that introduces a region of homozygosity.

The deletion region included 131 RefSeq genes, including seven genes annotated as disease-causing in OMIM: (1) *SOD1* encoding superoxide dismutase-1, (2) *MRAP* encoding melanocortin 2 receptor accessory protein, (3) *IFNAR2* encoding Interferon alpha, beta, and omega, receptor 2, (4) *CRFB4* encoding cytokine receptor, family II, member 4, (5) *IFNGR2* encoding interferon gamma receptor-2, (6) *KCNE1* encoding a potassium voltage-gated channel, and (7) *RUNX1* encoding runt-related transcription factor 1.

Lyle and colleagues described 13 cases of partial monosomy 21, noting that most deletions occurred in proximal or distal 21q but not in a central region from physical position ~30–36 Mb (Fig 4B) [28]. They proposed that the phenotypic consequence of deletions in this central region would be too severe to be compatible with survival. We and others described additional 21q segmental monosomy cases that partially overlap this central region; additional examples have been reported in the DECIPHER database (Fig 4C–4E). The deletion region of proband 4 is notable because it is the only one to span this central region (Fig 4F). Furthermore, the deletion region contains a locus implicated in agenesis of the corpus callosum (OMIM 217990), potentially accounting for that condition in this individual.

## Discussion

The present study characterized the clinical and behavioral phenotypes of 14 children with autism and extreme behavioral problems including self-injury. A candidate genetic abnormality likely to be causal was identified in four of these cases, representing a prevalence of 29%. Relatively large (>100 kb) CNVs were present in all of the other 10 cases (S1 File), but their clinical significance was less clear. Diagnostic yield using genomic microarrays is typically 10–15% for patients from cohorts having intellectual disability and/or autism. However, the diagnostic yield for individuals displaying severe behavioral problems in addition to autism and intellectual disability has not been assessed. In the present study we studied trios as well as parent/child duos, including such pedigrees because often informed consent cannot be obtained from both parents of the families we enrolled. For five probands who were not part of trios we could not confirm CNVs were *de novo*.

Criteria for defining candidate chromosomal CNVs as potentially causal were based on conservative ISCA guidelines [8]. While these are candidates, the present findings are not able to indicate definitively that any of these variants is causal. For Probands 2 and 4, we observed large de novo CNVs (a duplication of 14 Mb and a hemizygous deletion of 6.5 Mb, respectively). Large CNVs tend to be clinically significant, yet may be benign [17]. For Proband 4, the likelihood of the CNV having a causal (clinically significant) role is greatly increased for two reasons. First, while segmental monosomy of chromosome 21 has been observed in dozens of cases, none spans this particular band (Fig 4F). Indeed, Lyle and colleagues suggested that deletions in this particular locus most likely preclude survival, with prevalence within the population approaching zero [28]. Second, the patient had agenesis of the corpus callosum, a phenotype that has been mapped to 21q22 (OMIM %217990)[29].

To gather evidence for a causal role, approaches that are likely to be useful include: (1) identifying additional patients having a similar clinical phenotype and an overlapping CNV, (2) characterizing the functional consequences of the variants in conserved syntenic regions in animal models, and (3) characterizing the biochemical and/or cellular consequences of the variant *in vitro*.

In this study we focused on CNVs larger than 20 kb. In addition to the 14 Mb and 6.5 Mb CNVs of Probands 2 and 4, we describe large CNVs of unknown significance including homozygous deletions (e.g. 128 kb in Proband 5), hemizygous deletions (e.g. 526 kb in Proband 12, 453 kb in Proband 11), and amplifications to copy number 4 (e.g. 177 kb in Proband 7) or copy number 3 (e.g. 1.54 Mb in Proband 13, 323 kb in Proband 8). Empirical results from studies of apparently normal individuals indicate that 1% to 2% of all CNVs are larger than 1 Mb (see [8]). In the Database of Genomic Variants (a catalog of human genome structural variation), 0.4% of CNVs are greater than 1 Mb [30]. Thus it is possible for such large CNVs to occur in the context of an apparently normal phenotype. On the other hand, large CNVs occurring in affected individuals are commonly assumed to have causal roles, particularly when they are recurrent.

### The relevance of CNVs to SIB, autism, intellectual disability, and other clinical phenotypes

The occurrence of autism together with SIB was an inclusion criterion for this study. Autism was diagnosed by clinical experts. Notably, it can be impractical to administer standard diagnostic tests for autism spectrum disorders such as the ADI-R or ADOS when patients have other impairments as severe as those of the current sample. For the CNVs described as potentially pathogenic in the present study, the relevance to SIB, autism spectrum disorders, and ID considered individually cannot be determined at this point. In the future, it may be possible to identify individuals having various subsets of these traits and then to perform genotyping to identify CNVs. Recurrent CNVs may be associated with particular clinical phenotypes. Smith-Magenis syndrome, caused by interstitial deletions on chromosome 17p11.2, provides an example of a syndrome involving self-injurious behavior, intellectual disability, and other features. Potocki-Lupski syndrome involves duplication of the same region on chromosome 17 and has overlapping clinical features. Genotype and phenotype may be correlated: Prader-Willi syndrome (OMIM #176270) is a condition associated with both a well-characterized chromosomal change (deletion of paternal copies the imprinted *SNRPN* gene and other genes in the 15q11-q13 chromosomal region) and a particular topography of severe self-injurious behavior (skin-picking). There is no reported heritability for SIB, although the genetics of SIB are poorly studied.

### Future directions

Whole exome or whole genome sequencing in this patient population is likely to increase the yield of variants contributing to the phenotypes. Exome sequencing studies suggest a 10% contribution from de novo single nucleotide variants [31–35] and an additional 5% contribution to ASD risk from homozygous or compound heterozygous loss-of-function variants or hemizygous X-chromosome knockouts in males [36]. Gilissen et al. [37] described a cohort of 50 patients with severe intellectual disability and their parents. These patients had not received a molecular diagnosis after both SNP arrays and exome sequencing. Additional CNVs as well as loss-of-function mutations were identified by whole genome sequencing of trios, producing a diagnostic yield of 42% (and an estimated 62% cumulative diagnostic yield including microarrays and sequencing). Thus it is likely that exome or genome sequencing will greatly increase the diagnostic yield of the cohort we are studying.

The approach we have described in this study involves detailed behavioral phenotyping of patients along with initial efforts to define the genotype. In the future, adopting this approach with a larger sample will enable us to assess the prevalence of genetic anomalies likely to be causal in this population. Defining genotypes associated with these severe phenotypes may lead to improved strategies for treatment depending on the nature of the molecular defects.

## Supporting Information

S1 File Summary of case reports and chromosomal findings based on SNP analyses for probands 5–14. (DOCX)

S1 Table Description of SIB topographies and percentage of participants who engaged in them. (DOCX)

S2 Table Percentage of probands who targeted specific body locations with SIB, percentage of probands who exhibited other problem behavior, and percentage of SIB topographies with a given functional analysis. (DOCX)
