# Supplementary material for: Copy Number Variants Associated with 14 Cases of Self-Injurious Behavior
Source: PLoS One. 2016 Mar 2;11(3):e0149646. doi: 10.1371/journal.pone.0149646 (PMC4774994; doi:10.1371/journal.pone.0149646)
Supplement: S1 File — (DOCX) [file pone.0149646.s001.docx]

**Supporting File 1.** Summary of case reports and chromosomal findings based on SNP analysis for Probands 5-14. We report all of these findings as negative because they do not meet our relatively conservative criteria for significance (see Materials and Methods, “Criteria for inclusion of candidate loci”). In particular these loci are excluded either because they are inherited from a parent or they do not contain genes of known clinical significance. Thus, it is possible that some of the observed anomalies that are currently of uncertain significance will need to be reclassified as clinically significant in the future.

**Proband 5: autism with SIB.** The proband was a 10 year old male diagnosed with ASD, stereotypic movement disorder with self-injury, and moderate intellectual disability. His SIB included head-hitting and self-biting.

SNP analyses of the proband and his mother revealed the following CNVs that were not inherited (Table S3). (1) A 128 kilobase homozygous deletion harboring no genes. (2) A 54 kb homozygous deletion including *RHD* implicated in blood type disorders. (3) A 31 kb homozygous deletion including *LCE3C* and a portion of *LCE3B* but no OMIM disease-associated genes. (4) A hemizygous deletion of 451 kb on chromosome 1 affecting no OMIM genes (and including *NBPF25P*, *FAM231D*, and *FCGR1C*). (5) A 135 kb hemizygous deletion on chromosome 6 affecting several *HLA* genes implicated in susceptibility to sarcoidosis (*HLA-DRB1*) and susceptibility to celiac disease (*HLA-DQA1*). (6) A 118 kb hemizygous deletion on chromosome 19 containing one gene (*ZNF826P*) that is not disease-associated. (7) A 91 kb deletion on chromosome 15 harboring no genes. (8) An 88 kb deletion on chromosome 8 harboring five genes, none disease-associated. (9) A 65 kb deletion on chromosome 1 harboring no disease-associated genes. (10) A 51 kb deletion on chromosome 8 harboring no disease-associated genes. (11) Amplifications on chromosomes 3 and 15, neither of which contains disease-associated genes.

**Table S3.** Chromosomal alterations detected using a SNP array for Proband 5 (not inherited from his mother based on SNP analysis). Markers refers to number of SNP markers in each region. Length is in base pairs. UCSC coordinates refers to genome coordinates (build GRCh36/hg18). The proband’s father did not participate in this study and therefore it is possible that some of these alterations were inherited from him.

| Markers | Length (bp) | Copy number | UCSC coordinates |
| --- | --- | --- | --- |
| 38 | 127,878 | 0 | chr9:44795733-44667855 |
| 29 | 53,858 | 0 | chr1:25519573-25465715 |
| 35 | 30,888 | 0 | chr1:150853218-150822330 |
| 168 | 450,593 | 1 | chr1:147654217-147203624 |
| 60 | 135,314 | 1 | chr6:32734596-32599282 |
| 68 | 117,946 | 1 | chr19:20508229-20390283 |
| 13 | 91,444 | 1 | chr15:19566875-19475431 |
| 39 | 88,120 | 1 | chr8:7262162-7174042 |
| 37 | 65,180 | 1 | chr1:246863836-246798656 |
| 20 | 50,711 | 1 | chr8:12356009-12305298 |
| 60 | 83,214 | 3 | chr15:23051021-22967807 |
| 51 | 38,853 | 3 | chr3:131310768-131271915 |

**Proband** **6: autism with SIB.** The proband was diagnosed with ASD, stereotypic movement disorder with self-injury, unspecified disturbance of conduct, and severe intellectual disability. He had a history of grand mal seizures. Problem behaviors included aggression, self-injury (head-banging, head-hitting, and self-biting), biting others, pica, and spitting.

Based on SNP analysis we identified one large hemizygous deletion (218 kb on chromosome 1q21.1 involving no RefSeq genes) in a region in which the father had anamplification. A series of smaller de novo CNVs all involved no genes (including hemizygous deletions on chromosomes 14q11.2 and 3p21.31, and amplifications on chromosomes 12p11.1 and 21q21.1. We observed a gain of 1.121 megabases on chromosome 15q11.2. This matched a comparable sized gain in an unaffected brother (1.383 Mb) and a smaller gain (476 kb) in the mother.

**Table S4.** Chromosomal alterations detected using SNP arrays for Proband 6 that were not present in either the mother or father. Copy number path refers to PennCNV output for the interpretation of copy number variants based on data for the proband and his parents. The copy number states are 1 (total copy number 0; deletion of two copies), 2 (total copy number 1; deletion of one copy), 3 (total copy number 2; normal state), 4 (total copy number 2; copy-neutral with loss of heterozygosity), 5 (total copy number 3; single copy duplication), and 6 (total copy number 4; double copy duplication).

| Markers | Length (bp) | Copy number | Copy number path | UCSC coordinates |
| --- | --- | --- | --- | --- |
| 127 | 217,554 | 1 | 321 | chr1:147308487-147526040 |
| 48 | 73,979 | 1 | 221 | chr14:21909856-21983834 |
| 24 | 47,565 | 4 | 224 | chr12:34403012-34450576 |
| 32 | 40,202 | 1 | 221 | chr3:46784392-46824593 |
| 26 | 23,257 | 3 | 223 | chr21:18979549-19002805 |

**Proband 7.** The proband was a 13 year old male diagnosed with ASD, stereotypical movement disorder with self-injury, disruptive behavior disorder (not otherwise specified, NOS), obsessive compulsive disorder, bipolar affective disorder, and unspecified level of intellectual disability. Behavioral problems included self-injurious behaviors (jaw-popping fist contact to chin, head banging with fists to side of head, self-biting targeted at wrist), aggressive behavior (mostly when blocked from a ritualistic behavior) and inappropriate urination.

SNP analysis showed that the patient had two large CNVs. A 177 kb amplification (with copy number 4) occurred on chromosome Xq27.2, overlapping the gene *SPANXA2* encoding an RNA. The mother had three copies in this region, and the father two. There was also a *de novo* homozygous deletion of 113 kb on 3q26.1, overlapping no genes. We detected three smaller CNVs (ranging from 31 kb to 44 kb). These included an amplification on 89p23.1 (including *FAM66A* and *DEFB109P1* encoding a defensin pseudogene), a homozygous deletion on 5q35.3 including a portion of *BTNL3* encoding butyrophilin-like 3, and an amplification on chromosome 1 (at a locus in which the father had a hemizygous deletion) involving *LCE3C* encoding late cornified envelope protein 3C.

**Table S5.** Proband 7 CNVs. Data are from the proband (rows) and with copy number path reflecting additional data from his parents (copy number paths are defined in the legend to Table S4).

| Markers | Length (bp) | Copy number | Copy number path | UCSC coordinates |
| --- | --- | --- | --- | --- |
| 77 | 176,650 | 4 | 234 | chrX:140434071-140610720 |
| 71 | 113,339 | 0 | 220 | chr3:163995351-164108689 |
| 43 | 44,919 | 3 | 223 | chr8:12260380-12305298 |
| 19 | 39,394 | 0 | 220 | chr5:180311316-180350709 |
| 33 | 30,752 | 3 | 223-023 | chr1:150822151-150852902 |

**Proband** **8.** The proband was a 12-year-old boy with ASD, stereotypic movement disorder with self-injury, disruptive behavior disorder (NOS), and previous multiple small bowel ulcers of unclear etiology. He had a history of aggressive behavior as well as head-banging and hitting, self-biting and scratching, and self-pinching. Following an endoscopy and colonoscopy he was diagnosed with gastroesophageal reflux disease (GERD) as well as lymphoid hyperplasia with possible Crohn's disease of the small bowel.

SNP results were obtained from the proband and his father. Three hemizygous deletions in the proband were in regions partially overlapping deletions in the father. Of CNVs not in the father, the largest (a 323 kb amplification) included the *NSF* gene that is required for vesicular trafficking, including synaptic vesicle-mediated neurotransmitter release. Additional de novo findings included (1) a 145 kb amplification on Xq21.31 involving no genes; (2) a 129 kb amplification on chromosome 12p13.31 involving a portion of the *SLC2A14* and *SLC2A3* genes encoding facilitated glucose transporters. The *SLC2A3* gene product, GLUT3, is a brain-type glucose transporter . (3) A 110 kb hemizygous deletion on 15q11.2 encompassing *CHEK2P2* encoding a pseudogene.(4) A 94 kb homozygous deletion on chromosome 17 spanning four genes, none of which is annotated as disease-causing in OMIM.

**Table S6.** De novo CNVs in Proband 8 that were not observed in the father. (Note that the unaffected mother’s genotype is unknown at this time and some of these alterations might be inherited from her.)

| Markers | Length (bp) | Copy number | UCSC Coordinates |
| --- | --- | --- | --- |
| 45 | 323,042 | 3 | chr17:41784437-42107479 |
| 46 | 145,275 | 2 | chrX:89607045-89752320 |
| 61 | 129,113 | 3 | chr12:7886054-8015167 |
| 30 | 109,857 | 1 | chr15:18728578-18838435 |
| 25 | 94,491 | 0 | chr17:18299671-18394162 |
| 53 | 92,265 | 3 | chr3:101832934-101925199 |
| 43 | 85,928 | 1 | chr1:12764515-12850443 |
| 29 | 74,608 | 2 | chrX:91147459-91222067 |
| 63 | 54,438 | 1 | chr4:2281-56719 |

**Proband 9.** The proband was a 12 year old Caucasian male with ASD, stereotypic movement disorder with self-injurious behavior, mixed expressive/receptive language disorder, and unspecified disturbance of conduct. He demonstrated mild self–injurious behaviors since he was approximately 6 years old; these behaviors primarily consisted of hand-to-chin tapping and hitting. Subsequently he began exhibiting more intense forms of self-injury (head-hitting, head-banging, leg-hitting, and hair-pulling), increased aggressive behavior (grabbing, scratching, kicking, kicking, and tearing clothes), obsessive-compulsive behaviors (e.g., pressing his chin and face against surfaces, licking the floor, crawling into small spaces), increased number of facial tics, loss of speech, increased frequency of body posturing, disruptive behavior (screaming, throwing objects, property destruction), and dangerous acts (lack of safety awareness, tantrums and SIB in moving vehicle).

Chromosomal analyses revealed an amplification of 118 kb on chromosome Xp11.4. In this region the father, mother, and proband had 2, 3 and 4 copies, respectively. This region includes the *ATP6AP2* gene encoding a renin receptor which is an accessory protein that associates with the transmembrane segment of lysosomal V-type ATPases. A mutation in this gene has been associated with intellectual disability and epilepsy (Ramser et al., 2005; OMIM #300423). D*e novo* CNVs included a homozygous deletion on chromosome 14q32.33 (involving no genes) and an amplification on X involving *CD99*, encoding a multifunctional cell surface glycoprotein.

**Table S7.** CNVs in Proband 9. Data are from a proband and his parents.

| Markers | Length (bp) | Copy number | Copy number path | UCSC coordinates |
| --- | --- | --- | --- | --- |
| 74 | 118391 | 4 | 234 | chrX:40257534-40375924 |
| 17 | 31,078 | 0 | 220 | chr14:105601409-105632486 |
| 36 | 31,068 | 3 | 223-203 | chr1:150822151-150853218 |
| 14 | 21,549 | 3 | 223 | chrX:2629654-2651202 |

**Proband 10.** The proband was a 13-year-old Caucasian female diagnosed with ASD, stereotypic movement disorder with self- injurious behavior, disruptive behavior disorder (NOS), mood disorder (NOS), profound intellectual disability, and recent onset of seizures. Her behaviors included aggression (especially pinching and biting), self –injury (head-hitting and banging, body-hitting, self-pinching, and eye-directed SIB), destructive behaviors, tantrums, and PICA.

SNP analysis was performed on the proband and both parents. This revealed two de novo CNVs: (1) a 389 kb deletion on chromosome 17q21 (a region including the *NSF* gene and *ARL17* encoding ADP-ribosylation factor-like 17 isoform b); and (2) a 70 kb deletion on chromosome 11p15.4 contained no annotated genes, flanked several hundred kilobases beyond the CNV by an uncharacterized long non-coding RNA (NR_047550.1) and an odorant receptor gene (*OR52B*).

**Table S8.** CNVs in Proband 10. Data are from a proband and her parents.

| Markers | Length (bp) | Copy number | Copy number path | UCSC coordinates |
| --- | --- | --- | --- | --- |
| 53 | 389,649 | 1 | 221 | chr17:41750187-42139835 |
| 25 | 69,702 | 0 | 110 | chr11:4206589-4276290 |

**Proband 11.**  The proband was a 6-year old male diagnosed with ASD, disruptive behavior disorder (NOS), stereotypic movement disorder with self-injurious behavior, and moderate intellectual disability. Behavioral problems include disruptive behavior (disruptions, tantrums, very hyperactive, self-stimulatory behaviors), SIB (head-hitting and banging), aggression (hitting, biting, kicking), and dangerous acts (eloping, lack of awareness of safety).

Previous genetic testing found a karyotype within normal limits. Other evaluations within normal limits included EEG, MRI of brain, and metabolic screen including urine organic acids.

We identified 12 CNVs that were not inherited from the proband’s mother (S9 Table). The largest of these included no OMIM disease genes, as follows: (1) a 453 kb deletion on 10q11.22 encompassing two RefSeq genes; (2) a 148 kb deletion on 15q11.2 including one gene (*LINC01193*); (3) a 101 kb amplification on 4p16.1 including two genes; (4) a 78 kb amplification on 6q14.1 containing no annotated genes; (5) a 58 kb deletion on 1q44 containing three provisional RefSeq genes; (6) a 48 kb deletion on 3p21.31 containing no annotated genes; and (7) a 29 kb homozygous deletion on 2p22.3 containing no annotated genes.

**Table S9.** CNVs in Proband 11. Data are from a proband and his mother. The proband’s father did not participate in this study and therefore it is possible that some of these alterations were inherited from him.

| Markers | Length (bp) | Copy number | UCSC coordinates |
| --- | --- | --- | --- |
| 119 | 452,912 | 1 | chr10:46669593-47122505 |
| 34 | 147,551 | 1 | chr15:19419324-19566875 |
| 69 | 100,568 | 3 | chr4:8979788-9080356 |
| 120 | 77,596 | 3 | chr6:79025784-79103380 |
| 32 | 58,346 | 1 | chr1:246798656-246857002 |
| 42 | 47,772 | 1 | chr3:46776821-46824593 |
| 37 | 45,646 | 1 | chr3:196914788-196960434 |
| 43 | 28,552 | 0 | chr2:34552819-34581371 |
| 27 | 27,440 | 1 | chr7:142176272-142203712 |
| 23 | 24,985 | 1 | chr12:11409688-11434673 |
| 44 | 24,322 | 4 | chr20:1508787-1533109 |
| 14 | 23,630 | 4 | chr19:59994795-60018425 |

**Proband 12.** The proband was a nine year old male who was diagnosed with ASD at age 3. Subsequent diagnoses included stereotypic movement disorder with self-injurious behavior, disruptive behavior disorder, mood disorder, and impulse control disorder. Behavioral problems included aggression (biting, pinching, pulling hair, kicking, throwing objects at others), self-injury (self-biting, hair-pulling, and self-hitting), disruptive behavior (dropping, disrobing, urinating on the floor, tantrums, elopement) and property destruction (throwing objects). Previous medical tests included EEG and Fragile X testing (both normal).

We identified no large, *de novo* CNVs and only one small deletion (24 kb on chromosome 6q16.3) involving no genes.

**Table S10.** CNVs in Proband 12. Data are from a proband and copy number path includes information from his parents.

| Markers | Length | Copy number | Copy number path | UCSC coordinates |
| --- | --- | --- | --- | --- |
| 41 | 526,374 | 1 | 211-201  inherited | chr9:44195483-44721856 |
| 126 | 188,042 | 3 | 243  inherited | chr17:41521621-41709662 |
| 190 | 163,449 | 3 | 423  inherited | chr10:47011195-47174643 |
| 42 | 157,713 | 3 | 243  inherited | chr7:143540168-143697880 |
| 10 | 74,407 | 1 | 021  inherited | chr17:40929917-41004323 |
| 113 | 66,121 | 1 | 101-201 | chr6:79025784-79091904 |
| 19 | 46,286 | 3 | 423  inherited | chr22:22680529-22726814 |
| 44 | 24,057 | 1 | 221 | chr6:103844669-103868725 |
| 9 | 21,443 | 3 | 243  inherited | chr2:97507179-97528621 |
| 32 | 21,060 | 1 | 201  inherited | chr11:5744656-5765715 |

**Proband 13.** The proband was a 17-year old African American male with ASD, disruptive behavior disorder (NOS), stereotypic movement disorder with self -injurious behavior, mood disorder (NOS), and severe intellectual disability). Medical problems include static encephalopathy of unknown etiology and GERD secondary to esophageal burn from chemical ingestion. Behavioral problems include SIB (head-htting, body-hitting, skin-picking, and body-slamming), disruptive behavior (disrobing, dropping, fecal smearing, ISB, noncompliance & medication refusal), aggression (hitting, kicking, punching, pushing people to ground, tried to assault sister with a knife), and dangerous acts (eloping, lack of awareness of safety). We obtained SNP data from the proband and his mother.

We analyzed CNVs in the proband and his mother and identified no likely causal variants. The five largest CNVs not shared with the mother were all amplifications in regions lacking OMIM disease-causing genes: (1) a 1.5 megabase amplification on 9p11.2 including seven RefSeq genes; (2) a 593 kb amplification on 8p23.1; (3) a 219 kb amplification on 21p11.2 (including a portion of *TPTE*); (4) a 196 kb amplification on 4p11 with no annotated genes; and (5) a 182 kb amplification on 8p23.1 with four RefSeq genes.

**Table S11.** CNVs in Proband 13. Data are from a proband and his mother. The proband’s father did not participate in this study and therefore it is possible that some of these alterations were inherited from him.

| Markers | Length | Copy number | UCSC coordinates |
| --- | --- | --- | --- |
| 135 | 1,540,067 | 3 | chr9:44795733-43255666 |
| 145 | 592,541 | 3 | chr8:7847316-7254775 |
| 43 | 219,281 | 3 | chr21:9978023-9758742 |
| 22 | 195,759 | 3 | chr4:49004611-48808852 |
| 64 | 182,128 | 3 | chr8:12487426-12305298 |
| 16 | 132,936 | 1 | chr1:12938082-12805146 |
| 15 | 116,875 | 3 | chr13:18041824-17924949 |
| 27 | 115,816 | 1 | chr15:19566875-19451059 |
| 71 | 113,338 | 0 | chr3:164108689-163995351 |
| 20 | 63,532 | 3 | chr11:4315864-4252332 |
| 94 | 56,931 | 3 | chr6:79082715-79025784 |
| 32 | 40,868 | 1 | chr17:19479350-19438482 |
| 18 | 36,867 | 3 | chr1:88465-51598 |
| 31 | 35,583 | 1 | chr16:62671716-62636133 |
| 55 | 33,569 | 1 | chr3:147145162-147111593 |
| 23 | 27,979 | 4 | chr12:8475939-8447960 |
| 11 | 26,864 | 1 | chr19:24279522-24252658 |
| 20 | 25,048 | 1 | chr5:17568801-17543753 |
| 49 | 23,809 | 3 | chr3:131282357-131258548 |

**Proband 14.** The proband was an 11 year-old male diagnosed with ASD, obsessive-compulsive disorder, moderate intellectual disability, and mood disorder (NOS). His SIB included self-biting, head-banging, and body-hitting.

We identified two large, de novo CNVs although neither had a suggestive causal role (based on the criteria described in Materials and Methods). (1) A 153 kb hemizygous deletion on 16p12.1, including a portion of one predicted RefSeq pseudogene. (2) A 151 kb hemizygous deletion on chromosome 8p11.23-p11.22 including ADAM metallopeptidase domain 3A (*ADAM3A*). A series of five smaller CNVs (25kb to 61 kb) including several predicted and provisional RefSeq genes, but none associated with a disease phenotype in OMIM.

**Table S12.** CNVs in Proband 14. Data are from the proband and distinct from either of his parents (ash shown in copy number path).

| Markers | Length | Copy number | Copy number path | UCSC coordinates |
| --- | --- | --- | --- | --- |
| 73 | 152,811 | 1 | 221 | chr16:22465433-22618243 |
| 58 | 151,363 | 1 | 201 | chr8:39354760-39506122 |
| 15 | 60,843 | 1 | 101-201 | chr12:9558717-9619559 |
| 20 | 56,731 | 1 | 221 | chr16:21444226-21500956 |
| 44 | 32,314 | 3 | 443-423 | chr20:1509580-1541893 |
| 16 | 29,639 | 3 | 423 | chr3:129867213-129896851 |
| 21 | 24,675 | 0 | 220 | chr8:12261219-12285893 |

**References**

Ramser, J., Abidi, F. E., Burckle, C. A., et al. A unique exonic splice enhancer mutation in a family with X-linked mental retardation and epilepsy points to a novel role of the renin receptor. Hum. Molec. Genet. 14: 1019-1027, 2005. PMID: 15746149
