# Supplementary material for: Copy Number Variants Associated with 14 Cases of Self-Injurious Behavior
Source: PLoS One. 2016 Mar 2;11(3):e0149646. doi: 10.1371/journal.pone.0149646 (PMC4774994; doi:10.1371/journal.pone.0149646)
Supplement: S1 Table — (DOCX) [file pone.0149646.s002.docx]

S1 Table. Description of SIB topographies and percentage of participants who engaged in them.

| SIB topography | Number of cases | Percent of cases |
| --- | --- | --- |
| Head-hitting (e.g., hitting head with open or closed fist or with an object from 6 inches or more) | 12 | 86 |
| Self-biting (e.g., closure of teeth around any area of the body) | 10 | 71 |
| Head-banging (e.g., hitting head to surfaces, including objects, furniture, walls, and floors, from a distance of 6 inches or more) | 9 | 64 |
| Body-hitting (e.g., hitting body with an open or closed fist and with one or two hands from 6 inches or more) | 7 | 50 |
| Self-scratching (e.g., moving fingernail across skin such that the skin turns red or breaks) | 4 | 29 |
| Self-pinching (e.g., grabbing skin from any part of body between two fingers and squeezing, pulling, or twisting) | 3 | 21 |
| Hand/wrist-banging (e.g., hitting any part of the back of the hand to a hard surface, including knuckles and wrists; does not include palm of the hand) | 2 | 14 |
| Chin-banging/pressing (e.g., hitting chin on hard surfaces) | 2 | 14 |
| Hair-pulling (e.g., closing hand around any amount of hair and pulling away from head in any direction) | 2 | 14 |
| Leg, foot, knee SIB (e.g., jumping up and landing on the knees on the ground) | 1 | 7 |
| Skin-picking (e.g., pressing or digging fingernail forcefully into skin of any part of body) | 1 | 7 |
| Eye-SIB (e.g., hitting, slapping, pressing with open or closed hand, finger, or knuckle to one or both eyes or any area between the bottom of nose to eyebrows) | 1 | 7 |
| Body-slamming (e.g., throwing entire body against a hard surface from distance of 12 inches or more) | 1 | 7 |
