# Supplementary material for: Copy Number Variants Associated with 14 Cases of Self-Injurious Behavior
Source: PLoS One. 2016 Mar 2;11(3):e0149646. doi: 10.1371/journal.pone.0149646 (PMC4774994; doi:10.1371/journal.pone.0149646)
Supplement: S2 Table — (DOCX) [file pone.0149646.s003.docx]

S2 Table. Percentage of probands who targeted specific body locations with SIB, percentage of probands who exhibited other problem behavior, and percentage of SIB topographies observed within each functional analysis condition.

| Body location | Number of probands | Percentage  of probands |
| --- | --- | --- |
| Head/face | 13 | 93% |
| Hand/finger/wrist/arm | 6 | 43% |
| Chin | 3 | 21% |
| Leg/knee/foot | 2 | 14% |
| Hair | 2 | 14% |
| Eye | 1 | 7% |
| Body unspecified | 11 | 79% |
| Other problem behavior |  |  |
| Aggression | 13 | 93% |
| Disruptive behavior | 13 | 93% |
| Dropping | 13 | 93% |
| Spitting | 1 | 7% |
| Elopement | 1 | 7% |
| Functional analysis | Number of topographies | Percentage of topographies (n=55) |
| Attention | 13 | 24% |
| Demand | 10 | 18% |
| Tangible-edible | 9 | 16% |
| Tangible-toy | 6 | 11% |
| Automatic | 23 | 42% |
| Multiply maintained | 18 | 33% |
| Insufficient data | 16 | 29% |
